# Individual versus Combinatorial Effects of Silicon, Phosphate, and Iron Deficiency on the Growth of Lowland and Upland Rice Varieties

**DOI:** 10.3390/ijms19030899

**Published:** 2018-03-18

**Authors:** Nanthana Chaiwong, Chanakan Prom-u-thai, Nadia Bouain, Benoit Lacombe, Hatem Rouached

**Affiliations:** 1 Agronomy Division, Department of Plant and Soil Sciences, Faculty of Agriculture, Chiang Mai University, Chiang Mai 50200, Thailand; nantana.c189@gmail.com; 2 BPMP, Université de Montpellier, CNRS, INRA, SupAgro, 2 Place Pierre Viala, 34060 Montpellier, France; nadia.bouain@gmail.com (N.B.); benoit.lacombe@supagro.fr (B.L.)

**Keywords:** silicon, phosphate, iron, rice ecotypes, mineral nutrient homeostasis

## Abstract

Mineral nutrient homeostasis is essential for plant growth and development. Recent research has demonstrated that the occurrence of interactions among the mechanisms regulating the homeostasis of different nutrients in plants is a general rule rather than an exception. Therefore, it is important to understand how plants regulate the homeostasis of these elements and how multiple mineral nutrient signals are wired to influence plant growth. Silicon (Si) is not directly involved in plant metabolism but it is an essential element for a high and sustainable production of crops, especially rice, because of its high content in the total shoot dry weight. Although some mechanisms underlying the role of Si in plants responses to both abiotic and biotic stresses have been proposed, the involvement of Si in regulating plant growth in conditions where the availability of essential macro- and micronutrients changes remains poorly investigated. In this study, the existence of an interaction between Si, phosphate (Pi), and iron (Fe) availability was examined in lowland (Suphanburi 1, SPR1) and upland (Kum Hom Chiang Mai University, KH CMU) rice varieties. The effect of Si and/or Fe deficiency on plant growth, Pi accumulation, Pi transporter expression (*OsPHO1;2*), and Pi root-to-shoot translocation in these two rice varieties grown under individual or combinatorial nutrient stress conditions were determined. The phenotypic, physiological, and molecular data of this study revealed an interesting tripartite Pi-Fe-Si homeostasis interaction that influences plant growth in contrasting manners in the two rice varieties. These results not only reveal the involvement of Si in modulating rice growth through an interaction with essential micro- and macronutrients, but, more importantly, they opens new research avenues to uncover the molecular basis of Pi-Fe-Si signaling crosstalk in plants.

## 1. Introduction

Silicon (Si) is the second most abundant element in soil and it is taken up by plant roots before being distributed to the different plant organs. Though it is not considered as an essential element for plant metabolism, it is recognized as an essential element for high and sustainable production of crops as it helps plants to overcome both abiotic and biotic stresses (e.g., prevention of lodging and insect infestation) [1,2]. Concerning the biotic response, it has been proposed that amorphous Si deposits in plant tissues could lead to increased rigidity and abrasiveness which reduce the plant’s digestibility to insect pests [3,4]. Si deposits were also proposed to induce specific plant chemical defenses, including phytohormone jasmonate signaling [5,6,7,8] and the release of plant volatiles [9]. Furthermore, it has been shown that Si application ameliorates plant growth and development during stress conditions (review, [10]). For instance, Si application appeared to enhance salt tolerance by improving root water uptake, decreasing ion toxicity (e.g., sodium), and increasing polyamine accumulation in cucumber [11]. Application of Si can also improve drought tolerance by enhancing root hydraulic conductance and water uptake in tomato [12]. A possible mechanism was proposed, in which Si mediates a decrease in the membrane oxidative damage, which may contribute to the enhancement of root hydraulic conductance [12]. Si has been shown to improve tolerance to the heavy metals (e.g., cadmium) in durum wheat by a localized protection mechanism consisting of armoring the root surface using Si-bearing compounds and limiting root-shoot translocation [13]. Furthermore, Si-mediated alleviation of aluminum (Al) toxicity by modulation of Al and Si uptake and antioxidant performance in ryegrass has been reported [14]. In the literature, the involvement of Si in regulating plant growth in conditions where the availability of essential macro- and micronutrients undergoes changes is consistently demonstrated. For example, it has been shown that Si alleviates iron (Fe) deficiency in cucumber by enhancing the mobilization of Fe in the root [15]. Nevertheless, the beneficial role of Si in plant nutrition deserves further attention.

In spite of the ubiquitous presence and effect of Si on different plant species, Si research has been performed mainly on monocots and, more particularly, in grasses [16]. This could be explained by the fact that grasses are high Si accumulators: for example, in rice (*Oryza sativa* L.), Si content may reach 10% of the total shoot dry weight [17,18]. It has been observed that supplying Si in the nutrient solution of rice resulted in an increase of shoot dry weight [19]. In addition to Si, rice growth and development are also known to depend on an adequate supply of both essential macro- and micronutrients, e.g., phosphorus (P) and Fe. P is a major component of biological molecules, including nucleic acids, energy sources, and phospholipids, and P content is in the range of 0.4–0.8% in rice dry matter, while P deficiency causes stunted growth in plants [20]. However, the beneficial effect of Si under P deficiency stress has been observed in many plants, including rice, barley, and maize, in the following manner: barley yield was higher in a field improved with Si than in a field without Si application when a P fertilizer was not applied [20]. On the other hand, Fe is an essential micronutrient for plant cells functioning, which plays a role as a cofactor in metabolic processes, especially during photosynthesis; therefore, it is not surprising to find that P or Fe deficiency inhibits plant growth. Many studies have analyzed the effects of P and/or Fe deficiency in rice at the molecular level. For example, a global gene expression analysis using microarray experiments was performed to investigate rice transcriptome alterations in response to Fe or P alterations and to explore the interactions between Fe and P signaling in rice [21]. The results of this study showed that the majority of transcript changes observed in the absence of Fe (−Fe growth conditions) were repressed if Pi was also absent (−Fe−Pi). At phenotypic level, it has been shown that Pi and Fe interact in an antagonistic manner to modulate rice growth [22,23,24]. For instance, rice growth is severely affected by Fe deficiency and can be inverted by simultaneous Fe and P deficiencies. In addition to rice, an interaction between P and Fe signaling pathways has been observed in many plant species and, so far, has been perhaps best studied in the model plant *Arabidopsis thaliana* (review, [25]). Whether phosphate (Pi) and Fe availability affects Si accumulation in rice remains unclear, and whether this phenomenon varies between lowland and upland rice varieties awaits examination; vice versa, whether Si availability affects macronutrient accumulation is, as well, poorly understood. In this study, the existence and the possible influence of the interactions between Si, Pi, and Fe on the shoot and root biomass in two rice varieties, i.e., lowland rice (SPR1) and upland rice (KH CMU), were assessed. The two rice varieties were selected for analysis in this study because of their different original ecotype systems. The upland rice is commonly grown in aerobic conditions, whereas the lowland rice is grown in flooded conditions; therefore, each rice type has adapted to the fluctuating macro- and micronutrient availability in its own ecosystem. Our phenotypic analysis revealed an interesting interplay between the three elements Pi, Fe, and Si, indicating that this interplay influences the growth of the two rice varieties in a contrasting manner. Our study thus paves the way for further genetic research work to uncover the molecular basis of Pi-Fe-Si interactions among rice varieties.

## 2. Results

### 2.1. The Effect of Silicon Deficiency on Rice Biomass Varies with the Availability of Pi and Fe

**Figure 1:**
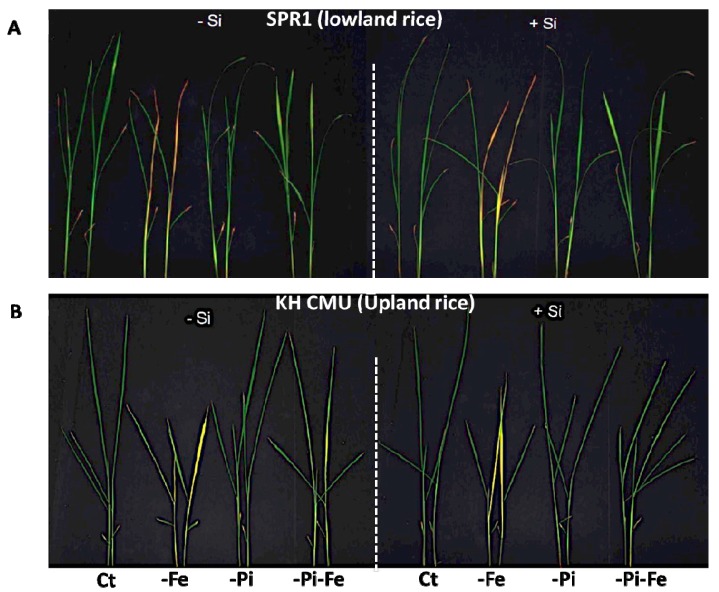
Phenotypes of lowland rice (SPR1) (**A**) and upland rice (KH CMU) (**B**) varieties grown under individual and combinatory nutrient deficiency conditions. The seedlings were grown for 18 days under non-aerated conditions in culture solution with and without silicon application. The dash lines are separated between growing condition of without Si (−Si) and with silicon (+Si) in each rice variety.

**Figure 2:**
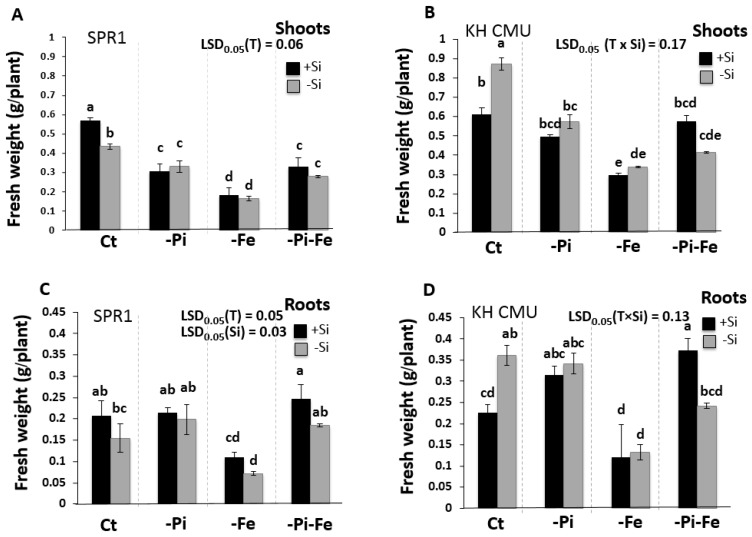
Fresh weight of lowland rice (SPR1) and upland rice (KH CMU) varieties grown under individual and combinatory nutrient deficiency stresses. The seedlings were grown in complete Yoshida media (CT), under single (−Pi and −Fe) or double (−Pi−Fe) nutrient deficiency stresses, with (+Si) and without (−Si) Si application. Shoot (**A**) and root (**C**) fresh weight of SPR1 and shoot (**B**) and root (**D**) fresh weight of KH CMU. The bars represent the standard errors of the corresponding means of the three replicates. Different lowercase letters indicate significant differences at *p* < 0.05. LSD, least significant difference.

In order to assess the effect of Si deficiency on the shoot and the root biomass of rice, two Thai rice varieties, namely, Suphanburi 1 (SPR1) and Kum Hom CMU (KH CMU), were grown hydroponically in the absence or in the presence of 1.5 mM of Si. Both varieties were grown for 18 days in a hydroponic system and produced a similar shoot and root biomass as the control treatment (Ct) (+Si) (Figure 1 and Figure 2). However, under Si deficiency (−Si), SPR1 showed 24% decrease in the shoot biomass in comparison to control growth conditions including Si (+Si) , but no difference was found in the root biomass, while KH CMU increased its biomass by 30% in the shoot and by 37% in the roots (Figure 2). These results revealed different responses to Si availability in the nutrient solution of the two rice varieties, with SPR1 being more sensitive to the lack of Si than the KH CMU variety.

It has been previously demonstrated that the shoot and root biomass of rice varieties are affected by the availability of Pi and/or Fe concentration in the medium [24]. Whether also an interaction among Si, Pi, and/or Fe homeostasis controls rice growth is unknown. Therefore, the effect of Pi and/or Fe deficiency on the shoot and root biomass of the two selected rice varieties, SPR1 and KH CMU, was assessed (Figure 1 and Figure 2). The rice varieties were grown in a hydroponic system for 18 days in the absence of Pi and/or Fe from the medium. The results showed that Pi deficiency affected the two varieties differently, with a significant decrease in the shoot biomass, but not in the roots, in SPR1, while there was no significant decrease in the biomass of KH CMU, both in the shoots and in the roots. A combination of Pi and Si deficiencies produced a similar effect as the one observed in the absence of Pi alone. Regardless of the presence or absence of Si, Fe deficiency caused a similar decrease of the biomass production of both varieties. Interestingly, the difference in the shoot biomass under Si deficiency in both varieties was maintained under a simultaneous Fe and Si deficiencies.

The remarkable capacity of rice plants to grow better under simultaneous Pi and Fe deficiencies compared to solely Pi or Fe stress appeared to be affected by the availability of Si in the growth medium. The simultaneous deficiencies of Si, Fe, and Pi did not affect the shoot biomass of both varieties compared to rice grown under Pi and Fe double deficiency stress, but they were found to cause a decrease of about 39% of the root biomass of KH CMU, while displaying no effect on that of SPR1. This result reveals a positive regulatory role of Si in the rice root biomass subjected to simultaneous Fe and Pi deficiency, especially in KH CMU.

### 2.2. The Availability of Pi and Fe Affects Si Accumulation in Rice

**Figure 3:**
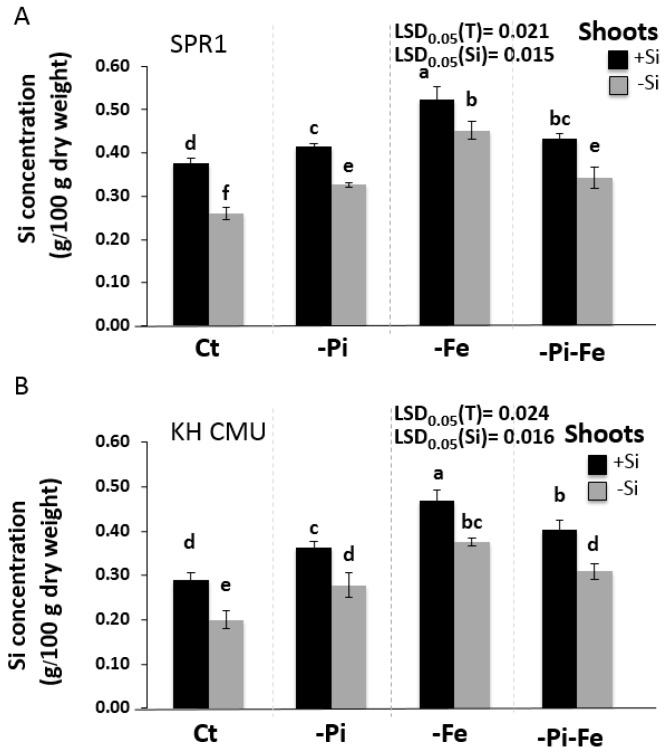
Silicon concentration in the shoot of lowland rice (SPR1) (**A**) and upland rice (KH CMU) (**B**) varieties grown under individual and combinatory nutrient deficiency stresses. The seedlings were grown for 18 days in complete Yoshida media (CT), under single (−P and −Fe) or double (−P−Fe) nutrient deficiency stresses, with (+Si) and without (−Si) Si application. The bars represent the standard errors of the corresponding means of the three replicates. Different lowercase letters indicate significant differences at *p* < 0.05.

The next objective was to assess the effects of Pi and/or Fe deficiency stress on Si accumulation in rice. The two varieties, SPR1 and KH CMU, were grown in the presence of Si and in the presence or absence of Pi and/or Fe (Figure 3). There were no significant differences in the Si concentration in the shoot between the varieties grown in the presence or absence of Si in the media. In the control condition, Si concentration in the roots in the presence of Si was found to increase by 42.6% and 73.7% in SPR1 and KH CMU, respectively, compared to that in the absence of Si. As expected, the deficiency of Si decreased Si accumulation in the tissue of the shoot in all the deficiency conditions in both varieties. However, in the presence of Si, but in the absence of Fe, Pi, or both Fe and Pi, both varieties accumulated higher Si in the shoot tissue than in the control condition (Figure 3). This positive effect of Fe deficiency on Si accumulation was diminished by the simultaneous stress of Si and Fe deficiency. In contrast, while both varieties accumulated similar Si concentrations under Pi and Fe deficiencies, this positive effect was cancelled to reach the control level under control conditions. These data constitute additional evidence for the interaction of Si, Pi, and Fe homeostasis in rice.

### 2.3. The Supply of Si and Fe Modulates Pi Accumulation in Rice

**Figure 4:**
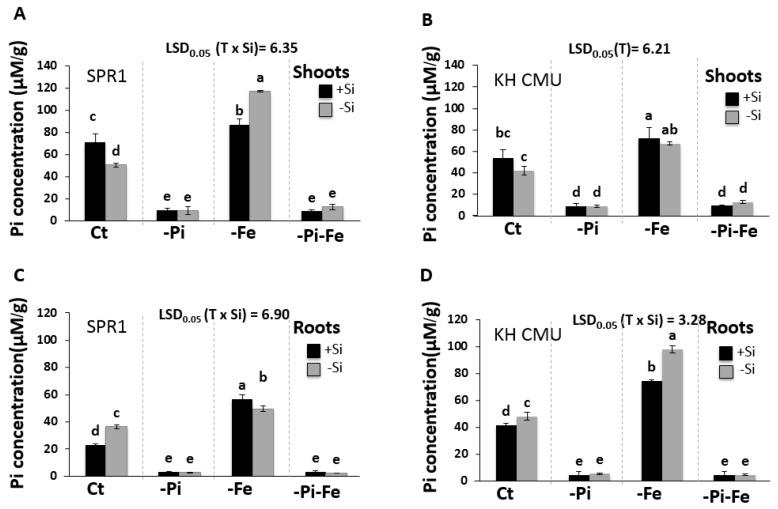
Phosphate concentration in lowland rice (SPR1) and upland rice (KH CMU) varieties grown under individual and combinatory nutrient deficiency stresses. The seedlings were grown for 18 days in complete Yoshida media (CT), under single (−P and −Fe) or double (−P−Fe) nutrient deficiency stresses, with (+Si) and without (−Si) Si application. Shoot (**A**) and root (**C**) Pi concentration of SPR1, and shoot (**B**) and root (**D**) Pi concentration of KH CMU. The bars represent the standard errors of the corresponding means of the three replicates. Different lowercase letters indicate significant differences at *p* < 0.05.

The effect of Si, Fe, and Pi availability on Pi accumulation in both varieties grown in a culture solution in the presence and the absence of 1.5 mM of Si, 40 µm of Fe, and/or 0.3 mM of Pi was tested (Figure 4). It is worth mentioning that the SPR1 variety accumulated significantly more Pi in the shoot compared to the KH CMU variety when grown in the presence of Si. Nevertheless, in the absence of Si, both varieties presented the same Pi concentration in the shoot. This indicates that Si deficiency caused a decrease in Pi accumulation only in the shoots of SPR1. Next, the effects of Si supply on Pi accumulation in the roots of the two varieties were determined. For SPR1 and KH CMU, the results showed that Si deficiency caused an increase in Pi accumulation in the roots. This increase was greater in SPR1 compared to KH CMU. As expected, combined Si and Pi deficiency reduced drastically Pi accumulation both in the shoot and in the root of both varieties compared to Si deficiency in the presence of Pi (Figure 4).

It is well established that Fe deficiency increases Pi accumulation in rice [24]. The results of this study confirmed the previous results, showing that Fe deficiency does increase Pi concentration in the shoots and the roots of SPR1 and KH CMU [24]. This increase was more pronounced in the roots of the two varieties than in the shoots. Regardless of the presence or absence of Si, simultaneous Pi and Fe deficiency was observed to reduce Pi concentrations in the shoot and the root of SPR1 and KH CMU. However, in the presence of Pi, simultaneous Fe and Si deficiency was observed to further increase the Pi concentration in the shoots of SPR1. In contrast, the positive effect of Fe deficiency alone on Pi concentration in the shoots of KH CMU was canceled by additional Si stress (stress due to simultaneous deficiency of Si and Fe). Remarkably, the opposite was observed in the roots. Simultaneous deficiency of Si and Fe was found to cause an increase of Pi accumulation in the roots of KH CMU but not in the roots of SPR1, where Pi concentration remained unchanged. Taken together, these data show that Si interacts with Fe to modulate the accumulation of Pi in rice and that SPR1 and KH CMU activate divergent molecular mechanisms in response to different Fe and Si supply, which distinctly affect Pi concentration.

### 2.4. The Availability of Pi and Si Affects Fe Accumulation in Rice

**Figure 5:**
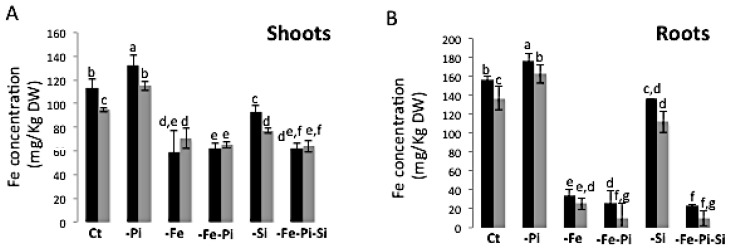
Iron concentration in the shoot (**A**) and roots (**B**) of lowland rice (SPR1) and upland rice (KH CMU) varieties grown in individual and combinatory nutrient deficiency stresses. The seedlings were grown for 18 days in complete Yoshida media (CT), under single (−P and −Fe) or double (−P−Fe) nutrient deficiency stress, with (+Si) and without (−Si) Si application (−Fe−Pi−Si). Shoots and roots were collected separately. Fe concentration was determined as described in [22] and presented as mg/kg DW. DW, dry weight. The black and grey histograms represent SPR1 and KH CMU, respectively. The bars represent the standard errors of the corresponding means of the three replicates. Different lowercase letters indicate significant differences at *p* < 0.05.

The effects of a combined Pi, Si, and/or Fe deficiency stress on the accumulation of Fe were assessed in the two rice varieties, SPR1 and KH CMU. As expected, −Pi increased Fe in the shoots and roots of both rice varieties [22]. −Fe or −Fe−Pi treatments caused a similar decrease in Fe concentration in the shoots and roots of SPR1 and KH CMU rice plants. Interestingly, under −Si conditions and despite the presence of Fe in the medium, Fe concentration in the shoots and roots of both rice varieties showed a significant decrease (Figure 5). These results are in agreement with a previous report showing that the leaf Fe concentration of plants in the presence of Si was >50% higher than in the absence of Si [15]. Triple nutrients deficiency stress, i.e., −Pi−Fe−Si, showed a similar effect on Fe concentration as observed by single Fe deficiency or simultaneous Pi and Fe deficiencies.

### 2.5. Si and Fe Homeostases Interact and Differentially Modulate Pi Root-to-Shoot Transfer in the Two Rice Varieties

**Figure 6:**
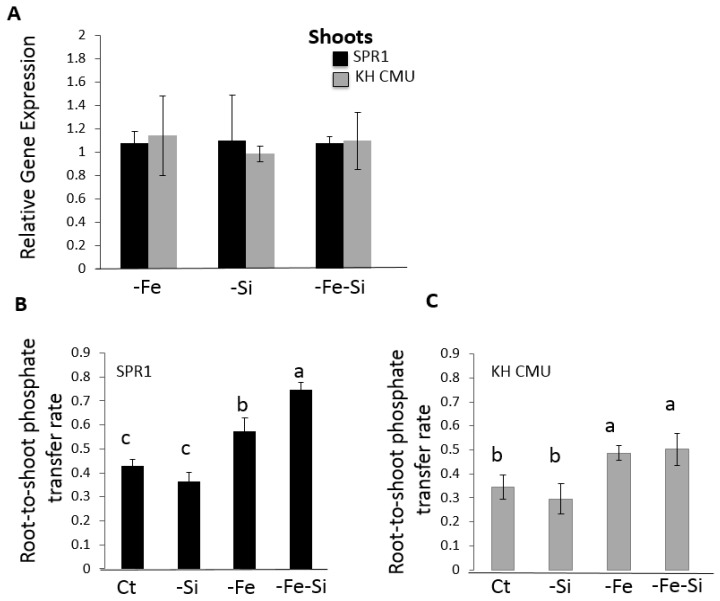
Expression of *OsPHO1;2* and effect of Fe and/or Si deficiency on the phosphate root-to-shoot transport in rice. (**A**) Rice seedlings were grown in Yoshida medium with different ionic concentrations: (**B**,**C**) rice varieties SPR1 and KH CMU were grown in media containing inorganic phosphate (Pi) in the presence or in the absence of iron (Fe) and silicon (Si). Abbreviations: complete medium (Ct) medium lacking either an individual element, i.e., iron (Fe) or silicon (−Si), or both (−Fe−Si). Quantitative real-time PCR (qRT-PCR) was performed to analyze *OsPHO1;2* mRNA levels; *OsActin1* expression level was used as the internal reference. Pi root-to-shoot transfer is defined as the ratio of radioactive Pi in the shoot over total radioactive Pi in the plant. Individual measurements were obtained from the analysis of shoots and roots collected from a pool of “*n*” plants (*n* > 3). The bars represent the standard errors of the corresponding means of the three replicates. Different lowercase letters indicate a significant difference between the treatment conditions at *p* < 0.05.

As aforementioned, SPR1 rice plants exposed to Fe deficiency led to the over-accumulation of Pi in the shoot, and this effect was further enhanced by a simultaneous Si and Fe deficiency (Figure 3). In contrast, in KH CMU, while Fe deficiency led to Pi over-accumulation in the shoot, no effect was observed under simultaneous stress of Si and Fe. This difference in Pi accumulation pattern prompted us to analyze the expression of the gene involved in the transfer of Pi from the root to the shoot in rice, namely *OsPHO1;2*, which is mostly abundant in the roots [22,26]. The expression of *OsPHO1;2* was analyzed in the roots of 18-day-old plants of the two rice varieties subjected to −Fe, −Si, or −Si−Fe conditions. Intriguingly, the results showed that there were no significant changes in the accumulation of *OsPHO1;2* transcript in either rice variety in all stress conditions in comparison to the control condition (Figure 6A). These data further are corroborated by [27], who showed that the regulation of *OsPHO1;2* expression varies depending on Pi availability and occurs mostly at the post-transcriptional/protein level. Therefore, it was decided to perform a functional test by determining the dynamics of Pi transport in both rice varieties grown in the presence of 0.33 mM Pi, with or without Fe and/or Si, using radiolabeled ^33^Pi. The results showed that Fe deficiency caused an increase in the root-to-shoot transport of Pi in rice and that simultaneous Fe and Si deficiencies exerted different effects compared to Fe deficiency alone in the two varieties (Figure 6B,C). In SPR1, Fe and Si deficiency caused an increase in the Pi translocation capacity (Figure 6B); however, in KH CMU, Fe and Si deficiency did not increase Pi translocation compared with Fe deficiency alone (Figure 6C). These data indicate a possible differential regulation of the protein involved in modulating Pi root-to-shoot ratio in KH CMU in comparison to SPR1.

## 3. Discussion

Rice varieties are generally classified into upland and lowland rice on the basis of their original ecosystem. Upland rice is grown in an aerobic conditions which cause low availability of P and Fe [28,29]. Lowland rice is grown in flood-like conditions where oxygen (O_2_) becomes the limiting factor for root functions such as the uptake of ions [30,31]. Rice plants must adapt to the fluctuating macro- and micronutrient availability, such as that of Pi, Fe, and Si. The results of this study revealed contrasting behaviors in two varieties representing lowland (SPR1) and upland (KH CMU) rice, in terms of their response not only to a change in Si availability, but also to changes in Pi and Fe availabilities. The application of Si appeared to enhance the shoot and root growth of both varieties compared to Si deficiency, which is in agreement with an early study showing that supplying Si in the nutrient solution resulted in an increase in the shoot dry weight of rice [19]. Recent research on Pi, Si, and Fe nutrition has focused only on the regulation of the homeostasis of each individual element. This study revealed the effect of each element on the concentration of the others and the existence of a complex bi- and tripartite interaction in the homeostasis of these three nutrients, Pi, Si, and Fe. The results showed the beneficial effect of Si under Pi deficiency stress on the biomass of the two rice varieties, SPR1 and KH CMU. This is in line with a previous report which showed that Si has an effect on plant growth under Pi deficiency in many plant species, including barley and maize. A study [20] has shown that in the absence of a P fertilizer, barley yield production was higher in a field supplied with Si than in a field without Si application. It has been also proposed that the P content in plants could be controlled by the availability of Fe, and Si application is known to alleviate Fe deficiency in plants. For example, Si application was found to alleviate Fe deficiency symptoms in cucumber by increasing shoot and root biomass and chlorophyll content [15,32]. Applying Si has been shown to improve rice plant growth by increasing the activity of catalases and polyphenol oxidases known as superoxide dismutases in both the root and shoot under Fe deficiency [33]. This data showed that Fe deficiency enhances Si accumulation in rice. Our results showed that the absence of Si led to the reduction of Fe accumulation in rice, which is in agreement with previous work showing that a lack of Si induced a 50% reduction of Fe accumulation [15]. It has been proposed that Si might indirectly affect transcription factors leading to the transcriptional activation of genes associated with Fe deficiency responses, and that Si may modulate root acquisition of Fe at early stages of Fe deficiency stress, through the regulation of the expression of Fe-related genes such as *iron-regulated transporter 1*, *ferric reduction oxidase 2* [15]. The precise mechanism that regulates Fe uptake and transport in rice under Si deficiency awaits examination. Our study showed that both rice varieties accumulated similar Si concentration under Pi and Fe deficiencies. These results provide support for the existence of a tripartite interaction in Si, Pi, and Fe homeostasis in rice, and show that Si is involved in regulating plant growth by modulating the essential macro (P)-nutrient and micro (Fe)-nutrient content.

In rice, members of the *OSPHO1* gene family, namely, *OsPHO1;1*, *OsPHO1;2*, and *OsPHO1;3*, have been identified [26]. The major role of *OsPHO1;2* is in the transfer of Pi from the root to the shoot, which is regulated by Pi deficiency [26]. This result revealed that the transcript accumulation of *OSPHO1;2* remains unchanged in rice roots when grown in the absence of Si, Fe, or both. Nevertheless, an increase in the Pi translocation from root to shoot in −Fe and −Si−Fe conditions in comparison to the control condition was observed. The increase in Pi transfer was similar in −Si and −Si−Fe conditions in the KH CMU variety, whereas, in the SPR1 variety, Pi transfer was higher in −Si−Fe condition than in the absence of Fe alone. An attractive hypothesis to explain these results is that the well-described *cis*-*natural antisense* of *PHO1;2* (*cis-NATPHO1;2*), known to play a role in promoting *OsPHO1;2* translation [27], affects Pi homeostasis and plant fitness. Thus, the regulation of *OsPHO1;2* by its *cis-NATPHO1;2* should be further examined in this context. Furthermore, we hypothesize that the regulation of *OsPHO1;2* at the protein level could be a target of a signaling pathway activated by −Fe−Si deficiency. Genes involved in the regulation of Pi-homeostasis [34,35,36] and in the co-regulation of P and other micro-or macronutrients homeostasis, such as that of zinc [35,37] or sulfate [35,38], could also play a role. In addition, the two different rice ecotypes may respond differently to this complex P, Fe, and Si interaction in aerobic growing conditions with varying amount of oxygen. These growing conditions would significantly affect the availability of nutrients, a situation that was not the focus of this study and requires further investigation in the future.

## 4. Materials and Methods

### 4.1. Rice Growth Condition

The experiment was arranged in factorial of Completely Randomize Design with three independent replications. Two rice (*Oryza sativa* L.) varieties were used in this study (SPR1, lowland rice and KH CMU, upland rice). The plants were grown in a controlled chamber: relative humidity of 80% and 14 light/10 h dark cycle, 200 µmol photons·m^−2^·s^−1^, and temperature of 28/25 °C. The seeds were dehusked and soaked in deionized water overnight in the darkness. Then, sixteen seedlings per treatment were exposed to light for 2 days and transferred to one-fourth of the full-strength nutrient solution. The experiments were repeated three times. The full-strength nutrient solution was used for the control treatment (Ct). The composition of the nutrient solution at full concentration was as follows: NaH_2_PO_4_, 0.33 mM; MgSO_4_, 1.64 mM; NH_4_NO_3_, 1.43 mM; K_2_SO_4_, 0.51 mM; CaCl_2_, 0.75 mM; Fe-NaEDTA, 40 µM; H_3_BO_3_, 20 µM; MnCl_2_, 10 µM; ZnSO_4_, 2.5 µM; CuSO_4_, 0.16 µM; and (NH_4_)_6_Mo_7_O_24_, 0.08 µM (modified from Saenchai et al. [22] and Yoshida et al. [39]). The treatment including Si consisted of SiO_2_ at a concentration of 1.5 mM (+Si), while no SiO_2_ was added in the other treatment (−Si). In the nutrient solution, NaH_2_PO_4_ as Pi and Fe-NaEDTA as Fe were omitted for the single or the combined stress, i.e., −Pi, −Fe, and −P−Fe. For all these treatments, the solution contained 2.5 mM MES buffer and was renewed every 5 days; pH was adjusted to 6.5 using citric acid.

### 4.2. Sample Collection

The plant samples were separated into shoot and root parts at harvest (10 days after the treatments were applied). The fresh weights of the total shoots and roots were measured. Then, the samples were analyzed for nutrient concentration.

### 4.3. Nutrient Concentration Analysis

For phosphate (Pi) concentration, the samples were incubated in water for 3 h at 70 °C. The Pi measurements were performed as follows: The quantification of Pi was performed by the molybdate assay as described by Saenchai et al. [22] and according to Ames [40]. The analysis for Si concentration was conducted by using a spectrophotometer at 650 nm after digestion in 50% NaOH, using the method of Dai et al. [41]. Iron was quantified as described by [22].

### 4.4. Real-Time Quantitative Reverse-Transcription PCR

Frozen root tissues were used for DNA-free total RNA extraction using the Plant RNeasy extraction kit and RNAse-free DNAseI (SIGMA-ALDRCH, St. Louis, MO, USA). Total RNA (two micrograms) was reverse transcribed using ThermoTM script RT (Invitrogen, Waltham, MA, USA). The quantification of transcripts abundance was performed with a cycler (LightCycler^®^480; Roche Diagnostics, Basel, Switzerland). Complementary DNA (cDNA) was used for the different PCR reactions containing 12.5 μL of the LightCycler^®^480 SYBR Green I Master mix (Roche, Indianapolis, IN, USA), each of the forward and reverse primers for *OsPHO1;2* and *OsActin1* (used as the housekeeping gene), and 5 μL of a 1:50 cDNA dilution in a final volume of 25 μL, which were performed for gene expression analyses [22]. The PCR reactions were performed in triplicate, and the related transcript quantification was performed using the comparative control treatment method [42,43].

### 4.5. Phosphate Root-to-Shoot Translocation Measurements

Pi uptake and root-to-shoot transfer measurements were performed using the upland rice (KH CMU) and lowland rice (SPR1) varieties grown hydroponically, after germination stage, for 10 days, and for additional 10 days in under control condition, single nutrient deficiency of Fe or Si, or double nutrient deficiency of Fe and Si. For root-to-shoot translocation, the roots were placed in Na_2_PO_4_ solution at pH 5.0 in the presence of 10 µCi/mL of the radiotracer ^33^P-orthophosphoric acid for 2:30 h. The rice plants were then washed in an ice-cold 5 mM Na_2_PO_4_ solution; thereafter, the shoots and roots were collected separately before drying. The radioactivity was measured using liquid scintillation counting (Tri-Carb 2900TR; Packard Instrument, Meriden, CT, USA) [44,45]. The calculation of the percentage of radioactivity located in the shoot over the total amount of radioactivity in the whole rice plant allowed the determination of the root-to-shoot Pi translocation.

### 4.6. Statistical Analysis

Statistical analyses of the data were performed by using the Statistica 9 software (analytical software SX, version 9, Tallahassee, FL, USA). The analysis of variance (ANOVA) was used to detect the differences between the treatments, and the least significant difference (LSD) at *p* < 0.05 was used to compare the means. The significance of the correlation coefficients was analyzed by pearson correlations at *p* < 0.05.

## 5. Conclusions

This study revealed the involvement of Si in modulating rice growth through interactions with essential micro- and macronutrients. This information provides evidence for the existence of a genetic basis for the interaction of the three nutrients Pi, Si, and Fe in plants. In the future, it would be important to discover the key genes involved in the co-regulation of Pi, Si, and Fe homeostasis. This task could be facilitated by the use of genetic variations of rice, such as the SPR1 and KH CMU varieties, that show different responses to Pi, Si, and/or Fe deficiency stress. This type of studies is important to enable biotechnological and agronomic strategies aimed at enhancing the uptake of these nutrients and improving rice yield in P-, Si-, and/or Fe-deficient soils.

## References

[1] Reynolds O.L., Keeping M.G., Meyer J.H. (2009). Silicon-augmented resistance of plants to herbivorous insects: A review. Ann. Appl. Biol..

[2] Meharg C., Meharg A.A. (2015). Silicon, the silver bullet for mitigating biotic and abiotic stress, and improving grain quality, in rice?. Environ. Exp. Bot..

[3] Keeping M.G., Meyer J.H. (2006). Silicon-mediated resistance of sugarcane to *Eldana saccharina* Walker (Lepidoptera: Pyralidae): Effects of silicon source and cultivar. J. Appl. Entomol..

[4] Massey F.P., Hartley S.E. (2009). Physical defences wear you down: Progressive and irreversible impacts of silica on insect herbivores. J. Anim. Ecol..

[5] Fawe A.B., Abou-Zaid M., Menzies J.G., Bélanger R.R. (1998). Silicon-mediated accumulation of flavonoid phytoalexins in cucumber. Phytopathology.

[6] Gomes F.B., Moraes J.C., Santos C.D., Goussain M.M. (2005). Resistance induction in wheat plants by silicon and aphids. Sci. Agric..

[7] Fauteux F., Chain F., Belzile F., Menzies J.G., Bélanger R.R. (2006). The protective role of silicon in the Arabidopsis-powdery mildew pathosystem. Proc. Natl. Acad. Sci. USA.

[8] Ye M., Song Y.Y., Long J., Wang R.L., Baerson S.R., Pan Z.Q. (2013). Priming of jasmonate-mediated antiherbivore defense responses in rice by silicon. Proc. Natl. Acad. Sci. USA.

[9] Kvedaras O.L., An M., Choi Y.S., Gurr G.M. (2010). Silicon enhances natural enemy attraction and biological control through induced plant defenses. Bull. Entomol. Res..

[10] Kim Y.H., Khan A.L., Waqas M., Lee I.J. (2017). Silicon Regulates Antioxidant Activities of Crop Plants under Abiotic-Induced Oxidative Stress: A Review. Front. Plant Sci..

[11] Wang W., Sardans J., Lai D.Y.F., Wang C., Zeng C., Tong C., Liang Y., Penuelas J. (2015). Effects of steel slag application on greenhouse gas emissions and crop yield over multiple growing seasons in a subtropical paddy field in China. Field Crops Res..

[12] Shi Y., Zhang Y., Han W., Feng R., Hu Y., Guo J., Gong H. (2016). Silicon enhances water stress tolerance by improving root hydraulic conductance in *Solanum lycopersicum* L.. Front. Plant Sci..

[13] Rizwan M., Meunier J.D., Davidian J.C., Pokrovsky O.S., Bovet N., Keller C. (2016). Silicon alleviates Cd stress of wheat seedlings (*Triticum turgidum* L. cv. Claudio) grown in hydroponics. Environ. Sci. Pollut. Res..

[14] Pontigo S., Godoy K., Jiménez H., Gutiérrez-Moraga A., de la Luz Mora M., Cartes P. (2017). Silicon-mediated alleviation of aluminum toxicity by modulation of Al/Si uptake and antioxidant performance in ryegrass plants. Front. Plant Sci..

[15] Pavlovic J., Samardzic J., Maksimović V., Timotijevic G., Stevic N., Laursen K.H., Hansen T.H., Husted S., Schjoerring J.K., Liang Y. (2013). Silicon alleviates iron deficiency in cucumber by promoting mobilization of iron in the root apoplast. New Phytol..

[16] Deshmukh R.K., Ma J.F., Belanger R. (2017). Role of Silicon in Plants. Front. Plant Sci..

[17] Epstein E. (1999). Silicon. Ann. Rev. Plant Physiol. Plant Mol. Biol..

[18] Ma J.F., Yamaji N. (2006). Silicon uptake and accumulation in higher plants. Trends Plant Sci..

[19] Ma J.F., Takahashi E. (1990). The effect of silicic acid on rice in a P-deficient soil. Plant Soil..

[20] Kewai H., Yan L., Guan L.Z. (2002). Effect of supply silicon on adsorption and desorption action of phosphorus in paddy soil. Plant Nutr. Fert. Sci..

[21] Zheng L., Huang F., Narsai R., Wu J., Giraud E., He F., Cheng L., Wang F., Wu P., Whelan J. (2009). Physiological and transcriptome analysis of iron and phosphorus Interaction in rice seedlings. Plant Physiol..

[22] Saenchai C., Bouain N., Kisko M., Prom-u-thai C., Doumas P., Rouached H. (2016). The involvement of *OsPHO1;1* in the regulation of iron transport through integration of phosphate and zinc deficiency signaling. Front. Plant Sci..

[23] Rouached H., Rhee S.Y. (2017). System-level understanding of plant mineral nutrition in the big data era. Curr. Opin. Syst. Biol..

[24] Mongon J., Chaiwong N., Bouain N., Prom-u-Thai C., Secco D., Rouached H. (2017). Phosphorus and Iron Deficiencies Influences Rice Shoot Growth in an Oxygen Dependent Manner: Insight from Upland and Lowland Rice. Int. J. Mol. Sci..

[25] Briat J., Rouached H., Tissot N., Gaymard F., Dubos C. (2015). Integration of P, S, Fe and Zn nutrition signals in Arabidopsis thaliana: Potential involvement of PHOSPHATE STARVATION RESPONSE1 (PHR1). Front. Plant Sci..

[26] Secco D., Baumann A., Poirier Y. (2010). Characterization of the rice PHO1 gene family reveals a key role for *OsPHO1;2* in phosphate homeostasis and the evolution of a distinct clade in dicotyledons. Plant physiol..

[27] Jabnoune M., Secco D., Lecampion C., Robaglia C., Shu Q., Poirier Y. (2013). A rice cis-natural antisense RNA acts as a translational enhancer for its cognate mRNA and contributes to phosphate homeostasis and plant fitness. Plant Cell..

[28] Yoshida S. (1981). Fundamentals of Rice Crop Science.

[29] Ponnamperuma F.N. (1975). Growth-limiting factors of aerobic soils. Major Research in Upland Rice.

[30] Drew M.D., Mullins A.P., Rice D.A. (1994). Synthesis, characterization and structural properties of some copper (II) trans-cinnamates and related compounds. Polyhedron.

[31] Mongon J., Jantasorn A., Oupkaew P., Prom-u-Thai C., Rouached H. (2017). The Time of Flooding Occurrence is Critical for Yield Production in Rice and Vary in a Genotype-Dependent Manner. Online J Biol Sci..

[32] Bityutskii N., Pavlovic J., Yakkonen K., Maksimović V., Nikolic M. (2014). Contrasting effect of silicon on iron, zinc and manganese status and accumulation of metal-mobilizing compounds in micronutrient-deficient cucumber. Plant Physiol. Biochem.

[33] Pavlovic J., Samardzic J., Kostic L., Laursen K.H., Natic M., Timotijevic G., Schjoerring J.K., Nikolic M. (2016). Silicon enhances leaf remobilization of iron in cucumber under limited iron conditions. Ann. Bot..

[34] Secco D., Bouain N., Rouached A., Prom U.T.C., Hanin M., Pandey A.K., Rouached H. (2017). Phosphate, phytate and phytases in plants: From fundamental knowledge gained in Arabidopsis to potential biotechnological applications in wheat. Crit. Rev. Biotechnol..

[35] Pal S., Kisko M., Dubos C., Lacombe B., Berthomieu P., Krouk G., Rouached H. (2017). TransDetect identifies a new regulatory module controlling phosphate accumulation in Arabidopsis. Plant Physiol..

[36] Bouain N., Doumas P., Rouached H. (2016). Recent Advances in Understanding the Molecular Mechanisms Regulating the Root System Response to Phosphate Deficiency in Arabidopsis. Curr. Genom..

[37] Kisko M., Bouain N., Safi A., Medici A., Akkers R.C., Secco D., Fouret G., Krouk G., Aarts M.G., Busch W. (2018). LPCAT1 controls phosphate homeostasis in a zinc-dependent manner. eLife.

[38] Rouached H. (2011). Multilevel coordination of phosphate and sulfate homeostasis in plants. Plant Signal Behav..

[39] Yoshida S., Foorno D.A., Cock J.H., Gomez K.A. (1976). Laboratory Manual for Physiological Studies of Rice.

[40] Ames B.N. (1966). Assay of inorganic phosphate, total phosphate and phosphatases. Methods Enzymol..

[41] Dai W.M., Zhang K.Q., Duan B.W., Sun C.X., Zheng K.L., Cai R., Zhuang J.Y. (2005). Rapid determination of silicon content in rice. Rice Sci..

[42] Livak K.J., Schmittgen T.D. (2001). Analysis of relative gene expression data using real-time quantitative PCR and the 2^−ΔΔ*C*t^ method. Methods..

[43] Rouached H., Wirtz M., Alary R., Hell R., Arpat A.B., Davidian J.C., Fourcroy P., Berthomieu P. (2008). Differential regulation of the expression of two high affinity sulfate transporters, SULTR1.1 and SULTR1.2, in Arabidopsis. Plant Physiol..

[44] Krouk G., Lacombe B., Bielach A., Perrine-Walker F., Malinska K., Mounier E., Hoyerova K., Tillard P., Leon S., Ljung K., Zazimalova E. (2010). Nitrate-regulated auxin transport by NRT1.1 defines a mechanism for nutrient sensing in plants. Developmental cell..

[45] Bouain N., Kisko M., Rouached A., Dauzat M., Lacombe B., Belgaroui N., Ghnaya T., Davidian J.C., Berthomieu P., Abdelly C. Phosphate/zinc interaction analysis in two lettuce varieties reveals contrasting effects on biomass, photosynthesis, and dynamics of Pi transport. Bio. Med. Res. Int..

